# Supplementary material for: Point prevalence of asymptomatic Plasmodium infection and the comparison of microscopy, rapid diagnostic test and nested PCR for the diagnosis of asymptomatic malaria among children under 5 years in Ghana
Source: PLoS One. 2020 Jul 27;15(7):e0232874. doi: 10.1371/journal.pone.0232874 (PMC7384639; doi:10.1371/journal.pone.0232874)
Supplement: S1 File — (DOCX) [file pone.0232874.s001.docx]

| Name & Contributions | Email | Affiliation |
| --- | --- | --- |
| **Bismark Okyere**  Contributions   - *Data Curation* - *Investigation* - *Validation* - *Writing – Original Draft Preparation* - *Writing – Review & Editing* | [bisokyere7@gmail.com](mailto:bisokyere7@gmail.com) | 1 |
| **Alex Owusu-Ofori**  Contributions   - *Conceptualization* - *Methodology* - *Supervision* - *Writing – Review & Editing* | [owusu_ofori@yahoo.com](mailto:owusu_ofori@yahoo.com) | 1 |
| **Daniel Ansong**  Contributions   - *Conceptualization* - *Methodology* - *Supervision* - *Writing – Review & Editing* | [ansongd@yahoo.com](mailto:ansongd@yahoo.com) | 2 |
| **Rebecca Buxton**  Contributions   - *Conceptualization* - *Methodology* - *Supervision* - *Writing – Review & Editing* | [rebecca.buxton@path.utah.edu](mailto:rebecca.buxton@path.utah.edu) | 3 |
| **Scott Benson**  Contributions   - *Conceptualization* - *Methodology* - *Investigation* - *Supervision* - *Writing – Review & Editing* | [scott.benson@hsc.utah.edu](mailto:scott.benson@hsc.utah.edu) | 4 |
| **Alex Osei-Akoto**  Contributions   - *Conceptualization* - *Methodology* - *Supervision* - *Writing – Review & Editing* | [alexosei_akoto@yahoo.com](mailto:alexosei_akoto@yahoo.com) | 2 |
| **Eddie-Williams Owiredu**  Contributions   - *Formal Analysis* - *Visualization* - *Writing – Original Draft Preparation* - *Writing – Review & Editing* | [eddiewilliams.owuredu@gmail.com](mailto:eddiewilliams.owuredu@gmail.com) | 5 |
| **Collins Adjei**  Contributions   - *Data Curation* - *Investigation* | [collins200889@gmail.com](mailto:collins200889@gmail.com) | 1 |
| **Evans Xorse Amuzu**  Contributions   - *Data curation* - *Investigation* - *Methodology* - *Project Administration* - *Writing – Review & Editing* | [evansxamuzu@gmail.com](mailto:evansxamuzu@gmail.com) | 6 |
| **Joseph Marfo Boaheng**  Contributions   - *Conceptualization* - *Investigation* - *Methodology* - *Project Administration* - *Writing – Review & Editing* | [marfoboaheng@yahoo.com](mailto:marfoboaheng@yahoo.com) | 6 |
| **Ty Dickerson**  Contributions   - *Conceptualization* - *Methodology* - *Supervision* - *Writing – Review & Editing* | [ty.Dickerson@hsc.utah.edu](mailto:ty.Dickerson@hsc.utah.edu) | 7 |

**^1^** Department of Clinical Microbiology, School of Medicine and Dentistry, Kwame Nkrumah University of Science and Technology, Kumasi, Ghana

**^2^** Department of Child Health, School of Medicine and Dentistry, Kwame Nkrumah University of Science and Technology, Kumasi, Ghana

**^3^** Department of Pathology, Medical Laboratory Science Division, University of Utah School of Medicine, Salt Lake City, USA

**^4^** Department of Family and Preventive Medicine, Division of Public Health, University of Utah School of Medicine, Salt Lake City, USA

**^5^** Department of Molecular Medicine, School of Medicine and Dentistry, Kwame Nkrumah University of Science and Technology, Kumasi, Ghana

**^6^** Research and Development Unit, Komfo Anokye Teaching Hospital, Kumasi, Ghana

^7^ Department of Pediatrics, University of Utah School of Medicine, Salt Lake City, USA
