# Supplementary material for: Point prevalence of asymptomatic Plasmodium infection and the comparison of microscopy, rapid diagnostic test and nested PCR for the diagnosis of asymptomatic malaria among children under 5 years in Ghana
Source: PLoS One. 2020 Jul 27;15(7):e0232874. doi: 10.1371/journal.pone.0232874 (PMC7384639; doi:10.1371/journal.pone.0232874)
Supplement: Attachment — Submitted filename: Response to Reviewers 28March2020.docx [file pone.0232874.s003.docx]

The Editor,

PLOS ONE

Dear Sir/ Madam,

**RESPONSE TO REVIEWERS**

The authors appreciate the timely and scrupulous review of our manuscript (PONE-D-20-02713). The authors note seventeen (17) comments in total. Kindly find below the responses to the reviewers’ comments. Tracked changes have been employed to highlight manuscript texts revised per the reviewers’ recommendations. Revised text indicated in this response are in “quotation marks”.

Reviewer #1:

Line 65: there is no reference between brackets

**Response 4:** Reference #4 has been added to the appropriate text

Lines 120 -121: Have you collected any information about LLIN use?

**Response:** Thank you for your comment. Because objective of the study is to determine the prevalence of asymptomatic malaria infection and evaluate the performance of RDT, microscopy and PCR for malaria diagnosis, we did not collect other data such as use of LLIN. However, we have included it as a limitation of the study (lines 279-282).

“This study is however limited the fact that we used only a single brand of malaria RDT; the prevalence may not be the same when other commercially available test kits are used. Also, data on the use of long-lasting insecticidal nets was unavailable. Additionally, the study was conducted in a peri-urban setting and the findings may not be generalizable to other areas. Thus, we recommend that further studies be conducted in the larger population.”

Lines 160-161: the sentence ‘Only P. 161 falciparum and P. malariae were identified in this study. There were no mixed infections by nPCR’ should be moved to Results section.

**Response:** Thank you for your comment. The statement has been moved to the results section (lines 178-179)

“Only *P. falciparum* [parasite density= 1,1540 (2,000- 34,000) parasites/µL] and *P. malariae* [parasite density= 2,100 (1,270- 3,720) parasites/µL] were identified in this study. There were no mixed infections”

One question: The PCR you used cannot diagnose P. vivax. As you consider this PCR as the reference, I think that there is a little bias. Could you explain this choice?

**Response:** Thank you for your comment. The PCR primers were selected based on the occurrence/prevalence of the different species of Plasmodium in Ghana. It is known that there are currently five major clinically relevant Plasmodium species, namely, *P. falciparum, P. ovale, P. malariae, P. vivax* and *P. knowlesi*. However, only *P. falciparum, P. ovale, P. malariae* have been documented to occur in Ghana (<https://www.who.int/malaria/publications/country-profiles/profile_gha_en.pdf?ua=1/> and <https://www.severemalaria.org/countries/ghana> and <http://www.ghanahealthservice.org/downloads/GHS_Antimalaria_drug_policy.pdf> ). We did not include *P. vivax* because reports of its infection in the Ghana is rare.

Reviewer #2:

1. Title: Well written but it can be rephrased to give a clear message. The authors were assessing the prevalence of asymptomatic P. falciparum infection in paediatric population in Ghana, but also compared the diagnostic tools, so they should rephrase these two sentences in order to capture reader's attention just from the title.

**Response:** Thank you for your comment. We have rephrased the title to clearly highlight the prevalence and comparison of test methods (lines 1-3).

“Prevalence of asymptomatic Plasmodium infection and the comparison of microscopy, rapid diagnostic test and nested PCR for the diagnosis of asymptomatic malaria among children under 5 years in Ghana”

2. Background: The concept of malaria prevalence in endemic areas and in the country where the study was conducted needs to be thoroughly explained. The authors should clearly indicate the relevance of conducting this study. A number of this kind of studies have been conducted in Ghana, I suggest that, the authors should clearly show that the study was conducted in (Atwima Nwabiagya) Northern district in Ghana, not just Ghana in general.

**Response:** Thank you for your comment. The relevance of the study has been clarified and (Atwima Nwabiagya) Northern district as the study site has been highlighted (lines 96-101)

“Although a number of studies on asymptomatic malaria in older children have been conducted across Ghana [30-32] and children under 5 in neighboring African countries [33-35], there remains a dearth of published data on asymptomatic malaria in children under 5 years in Ghana, particular in the northern sectors of the country where adequate health facilities are wanting. This study assessed the prevalence of asymptomatic malaria infection and evaluated the performance of malaria RDT, light microscopy and nested PCR (nPCR) for the diagnosis of asymptomatic malaria infection in children under 5 years old in Atwima Nwabiagya North district, Ghana.

3. Results: The authors need to check their results the way they have reported them. What I know and from several reports, asymptomatic malaria occurs in children above 5 years of age and not less than 5 because of immunity. But their results indicate children of 1-2 years appear to be asymptomatic compared to other age groups. They need to justify this.

- Figures are also missing in the document that I review.

- Parasite density is also important to be shown

- There should be parasite density of the results obtained by microscopy. This will add value and will be a relevant information as well.

**Response:** Thank you very much for your comment. The data has been rechecked and the results remained the same. In this study, simple random sampling procedure was used to recruit asymptomatic children. The higher prevalence of asymptomatic malaria among 1-2 years old children could be due to the greater number/size of 1-2 year aged children obtained in this study. This could also highlight the distribution of asymptomatic malaria cases in children under 5 in the region. The figures were included during the submission. Parasite densities have been included (table 1 and lines 178-179).

“Only *P. falciparum* [parasite density= 1,1540 (2,000- 34,000) parasites/µL] and *P. malariae* [parasite density= 2,100 (1,270- 3,720) parasites/µL] were identified in this study. There were no mixed infections”

4. Discussion: this section is poorly written. There are some facts that need to be considered in order to reflect the rationale of the study and the findings. Authors should handle all issues raised and re-write this section accordingly.

**Response:** Thank you for your comment. The discussion section has been revised, taking into consideration all comments attached (line 216-227)

“Based on PCR, this study reports a high prevalence (36.8%) of asymptomatic Plasmodium infection among a paediatric population in the Atwima Nwabiagya North district of Ghana. Asymptomatic malaria in children under 5 years has been reported in some African countries [33-35]. In Ghana, Crookston, et al. [46] reported an asymptomatic malaria prevalence of 31.8% based on PCR among children less than five years of age in Kumasi, Ghana which is similar to our study finding. Other studies in Ghana such as those by Dinko et al. [31] in Kumasi and Danquah et al. [47] in northern Ghana reported asymptomatic malaria among older children. The prevalence found in the studies by Dinko et al. (76.6%) and Danquah et al. (89.7%) are higher compared to this study and the discrepancy may be linked to the fact that older children tend to be the major asymptomatic carriers of Plasmodium compared to younger children [47, 48], possibly as a result of protective immunity acquired over the years.

We also found asymptomatic malaria to be more prevalent among male children compared to females, similar to the findings of Golassa et al. in Ethiopia [29]. The higher prevalence of asymptomatic malaria among male children compared to females could be explained by the fact that, in Ghana, males are more exposed to both daytime and nighttime outdoor activities than females and thus, at higher risk of mosquito bites compared to females who are usually indoors …”

5. Study strength and limitations: This section is missing. They should have clearly pointed this out.

**Response:** Thank you for your comment. We have included a section for study strength and limitations (line 273-282)

“Study strengths and limitations

The strength of the study is in the reporting of the prevalence of asymptomatic malaria infection among children less than 5 years old in the northern sector of Ghana. We also highlight potential hotspots for malaria screening and treatment during national and regional malaria surveillance exercises. The study also corroborates previous reports on the usefulness of molecular detection methods for asymptomatic malaria diagnosis. We showed that, in the absence of PCR, RDT performs better in the diagnosis of asymptomatic malaria among children compared to microscopy.

This study is however limited the fact that we used only a single brand of malaria RDT; the prevalence may not be the same when other commercially available test kits are used. Also, data on the use of long-lasting insecticidal nets was unavailable. Additionally, the study was conducted in a peri-urban setting and the findings may not be generalizable to other areas. Thus, we recommend that further studies be conducted in the larger population.”

6. Other minor comments

The authors should check the language throughout the manuscript and correct typos. I have done my comments in the manuscript in track changes.

**Response:** Thank you very much for your comment. The language and typos have been reviewed and revised and all comments included as tracked changes have been addressed.

**Miscellaneous Author Responses:**

- The 2010 Population and Housing Census of Ghana is the latest published data. A newer census is scheduled to take place in 2020.
- The authors initially chose to use the term *point prevalence* for malaria as our study reports the prevalence of malaria cases among individual asymptomatic children at a single point in time. Whereas point prevalence addresses the question of whether a person currently has malaria, *period prevalence* addresses the question of whether that subject had malaria at any time during the period under investigation. Since the study did not test individual children for malaria at multiple times over a defined period of time, we are unable to report the period prevalence for asymptomatic malaria infection.

Thank you once again for the timely review and scrupulous of our manuscript. Looking forward to hear favorably from you.

Sincerely,

Ty Dickerson

**Corresponding author**
